# Machine learning based assessment of hoarseness severity: a multi-sensor approach centered on high-speed videoendoscopy

**DOI:** 10.3389/frai.2025.1601716

**Published:** 2025-06-05

**Authors:** Tobias Schraut, Anne Schützenberger, Tomás Arias-Vergara, Melda Kunduk, Matthias Echternach, Stephan Dürr, Julia Werz, Michael Döllinger

**Affiliations:** ^1^Division of Phoniatrics and Pediatric Audiology at the Department of Otorhinolaryngology, Head and Neck Surgery, University Hospital Erlangen, Friedrich-Alexander-Universität Erlangen-Nürnberg, Erlangen, Germany; ^2^Pattern Recognition Lab, Chair of Computer Science, Friedrich-Alexander-Universität Erlangen-Nürnberg, Erlangen, Germany; ^3^Department of Communication Sciences and Disorders, Louisiana State University, Baton Rouge, LA, United States; ^4^Division of Phoniatrics and Pediatric Audiology at the Department of Otorhinolaryngology, Head and Neck Surgery, University Hospital Munich, Ludwig-Maximilian-Universität München, Munich, Germany; ^5^Division of Phoniatrics and Pediatric Audiology at the Department of Otorhinolaryngology, University Hospital Regensburg, Universität Regensburg, Regensburg, Germany

**Keywords:** machine learning, deep learning, high-speed videoendoscopy, voice disorders, hoarseness, image processing, signal processing, feature selection

## Abstract

**Introduction:**

Functional voice disorders are characterized by impaired voice production without primary organic changes, posing challenges for standardized assessment. Current diagnostic methods rely heavily on subjective evaluation, suffering from inter-rater variability. High-speed videoendoscopy (HSV) offers an objective alternative by capturing true intra-cycle vocal fold behavior. Integrating time-synchronized acoustic and HSV recordings could allow for an objective visual and acoustic assessment of vocal function based on a single HSV examination. This study investigates a machine learning-based approach for hoarseness severity assessment using synchronous HSV and acoustic recordings, alongside conventional voice examinations.

**Methods:**

Three databases comprising 457 HSV recordings of the sustained vowel /i/, 634 HSV-synchronized acoustic recordings, and clinical parameters from 923 visits were analyzed. Subjects were classified into two hoarseness groups based on auditory-perceptual ratings, with predicted scores serving as continuous hoarseness severity ratings. A videoendoscopic model was developed by selecting a suitable classification algorithm and a minimal-optimal subset of glottal parameters. This model was compared against an acoustic model based on HSV-synchronized recordings and a clinical model based on parameters from other examinations. Two ensemble models were constructed by combining the HSV-based models and all models, respectively. Model performance was evaluated on a shared test set based on classification accuracy, correlation with subjective ratings, and correlation between predicted and observed changes in hoarseness severity.

**Results:**

The videoendoscopic, acoustic, and clinical model achieved correlations of 0.464, 0.512, and 0.638 with subjective hoarseness ratings. Integrating glottal and acoustic parameters into the HSV-based ensemble model improved correlation to 0.603, confirming the complementary nature of time-synchronized HSV and acoustic recordings. The ensemble model incorporating all modalities achieved the highest correlation of 0.752, underscoring the diagnostic value of multimodal objective assessments.

**Discussion:**

This study highlights the potential of synchronous HSV and acoustic recordings for objective hoarseness severity assessment, offering a more comprehensive evaluation of vocal function. While practical challenges remain, the integration of these modalities led to notable improvements, supporting their complementary value in enhancing diagnostic accuracy. Future advancements could include flexible nasal endoscopy to enable more natural phonation and refinement of glottal parameter extraction to improve model robustness under variable recording conditions.

## 1 Introduction

Functional or malregulative dysphonia (FD) refers to an impairment of voice production, characterized by limitations in vocal capacity and acute or persistent changes in voice quality. Its diverse genesis in the absence of primary morphological changes poses a challenge for standardized assessment, resulting in a lack of consensus on diagnostic criteria (Altman et al., 2005; Schneider-Stickler and Bigenzahn, 2013).

In contrast to organic dysphonia, which can typically be diagnosed based on characteristic structural changes of the vocal folds, functional voice disorders are assumed to result solely from pathologically altered vibration patterns of the vocal folds. Therefore, the visual evaluation of functional voice disorders requires a detailed examination of vocal fold behavior. However, since structural abnormalities are absent, a key step in the comprehensive assessment of FD is voice quality evaluation, where perceptual characteristics such as hoarseness serve as essential indicators of vocal impairment (Schneider-Stickler and Bigenzahn, 2013; Voigt et al., 2010).

According to the European Laryngological Society (ELS) and the American Speech-Language-Hearing Association (ASHA), a comprehensive clinical assessment of the voice typically includes acoustic and aerodynamic measurements, auditory-perceptual evaluation, subjective self-assessment, and videolaryngoscopy (Dejonckere et al., 2001; Patel et al., 2018).

Acoustic and aerodynamic measurements are usually performed by a speech therapist as part of a multidimensional voice examination. Several recordings of sustained vowels are analyzed to determine the voice range profile along with relevant acoustic and aerodynamic parameters such as jitter, shimmer, harmonics-to-noise ratio (HNR) and maximum phonation time.

Auditory-perceptual assessment of voice quality involves the use of standardized rating scales, such as the GRBAS or RBH scale. Here, an expert evaluates continuous speech (e.g., *Rainbow Passage*) according to several criteria: grade (G) or hoarseness (H), roughness (R), breathiness (B), asthenia (B), and strain (S). For each criterion, a score of 0 (normal), 1 (mild), 2 (moderate), or 3 (severe) is provided. The overall ratings are derived as $G=\max\left(R,\;B,\;A,\;S\right)$ for the GRBAS and $H=\max\left(R,\;B\right)$ for the RBH scale, respectively (Schneider-Stickler and Bigenzahn, 2013).

Patients’ subjective self-assessment is conducted through questionnaires designed to measure their perceived quality of life concerning voice and voice disorders. Common questionnaires include the Voice Handicap Index ( VHI ) and the Voice-Related Quality of Life ( VRQOL ) (Hogikyan et al., 2000; Jacobson et al., 1997).

Finally, visual examination of the vocal folds at rest and during phonation facilitates the etiologic diagnosis of voice disorders and allows for the observation of vocal fold behavior. Currently, the most widely used laryngeal imaging technique is videostroboscopy, which artificially reconstructs the glottal cycle by compiling images captured at different phases across consecutive cycles (Deliyski, 2016).

Current voice diagnostic methods rely heavily on subjective assessment criteria (Voigt et al., 2010). While objective parameters are incorporated into acoustic analysis to support diagnosis, auditory-perceptual evaluation by trained experts remains the gold standard for assessing acoustic voice quality (Schneider-Stickler and Bigenzahn, 2013). The same subjectivity applies to visual assessment, where physicians evaluate features such as glottal closure, regularity, and symmetry (Dejonckere et al., 2001). However, this process especially demands considerable expertise and is time-consuming, labor-intensive, and prone to inaccuracies due to the high volume of visual data. Subjective evaluations are also influenced by factors like the rater’s experience, fatigue, and perceptual bias, all of which have been shown to negatively affect both inter- and intra-rater reliability (Lu and Matteson, 2014). To address these limitations, researchers are seeking an objective, standardized procedure for voice assessment through quantitative analysis of video and audio data.

Beyond subjectivity, a key limiting factor in visual assessment lies in the nature of videostroboscopy, which reconstructs the glottal cycle under the assumption of periodic vocal fold vibration, making it unsuitable for analyzing intra- or inter-cycle variations. Consequently, videostroboscopy cannot provide a reliable assessment for dysphonic patients with unstable phonatory characteristics (Deliyski, 2016).

High-speed videoendoscopy (HSV) is a promising laryngeal imaging technique with the potential to supersede videostroboscopy. With its high frame rates (≥4,000 Hz), HSV does not rely on the assumption of periodicity, but instead captures the true intra-cycle vibratory behavior of the vocal folds. This enables the measurement of intra-cycle characteristics such as vocal fold regularity, symmetry, and glottal closure, as well as cycle-to-cycle variations of these features (Deliyski, 2016).

HSV allows for the reliable quantification and objective analysis of vocal fold behavior. Additionally, many HSV systems enable the simultaneous recording of the acoustic signal. Integrating time-synchronized acoustic and HSV recordings offers the potential for an objective visual and acoustic assessment of vocal function based on a single HSV examination (Deliyski and Hillman, 2010; Mehta et al., 2010).

In recent years, machine learning (ML) and deep learning (DL)-based approaches have gained attention in the pursuit of objective, standardized voice assessment. While many of these methods focus on conventional acoustic recordings, HSV recordings have received comparatively little attention. Most studies utilizing ML on HSV data have focused on detecting structural changes in the vocal folds (i.e., organic voice disorders) rather than quantitatively analyzing vocal fold behavior (Barlow et al., 2024).

Voigt et al. (2010) explored the use of HSV data by extracting two feature sets from 75 HSV recordings (25 healthy, 50 FD) of the sustained vowel /a/ to distinguish between normal and pathological voices. The first set consisted of 10 features derived from the glottal area waveform (GAW) – a function describing the glottal area over time – capturing glottal dynamics and perturbation. The second set comprised 12 features based on the contours of the phonovibrogram (PVG), an image capturing the spatio-temporal movement patterns of vocal fold activity. Using a support vector machine (SVM) with ten-fold cross-validation (CV), they obtained accuracies of up to 0.809 and 0.817 for GAW- and PVG-based features, respectively.

Schlegel et al. (2020b) combined 91 GAW- and PVG-based features describing pitch, perturbation, noise, glottal dynamics and symmetry, and PVG contours into a feature set. A subset of 13 relevant parameters was determined using correlation analysis and feature importance measures based on boosted decision stumps. Applying LogitBoost on 358 HSV recordings (225 healthy, 133 FD) of the sustained vowel /i/, they achieved an accuracy of 0.757 in a ten-fold CV.

Arias-Vergara et al. (2023) investigated the use of novel features derived from the Nyquist plot representation of the GAW. Using 66 HSV recordings (33 healthy, 33 FD) of the sustained vowel /i/, they extracted 20 Nyquist plot-based features and 110 GAW-based features describing pitch, perturbation, noise, glottal dynamics, mechanics, and symmetry. A subset of 30 relevant parameters was selected using perturbation feature importance. Classification with an SVM in an 11-fold CV achieved an accuracy of 0.820 in distinguishing normal from FD voices.

Döllinger et al. (2012) classified sustained phonations recorded during HSV into normal and pathological voices using linear discriminative analysis based on 10 acoustic perturbation and noise parameters. They achieved accuracies of 0.900 for males (30 healthy, 30 disordered) and 0.730 for females (43 healthy, 43 disordered), respectively.

In Schraut et al. (2025), we developed an acoustic model for hoarseness severity estimation using 617 sustained phonations recorded during HSV. A combination of filter and wrapper selection methods reduced 490 acoustic parameters to a subset of five relevant features. Logistic regression (LR) applied to this feature set yielded a classification accuracy of 0.742 and a correlation of 0.637 between model predictions and hoarseness rating on a hold-out test set of 124 recordings.

So far, the use of HSV and acoustic recordings for ML-based voice assessment has only been explored independently. Studies investigating the relationship between acoustic and HSV-derived parameters reveal minimal redundancy between these modalities, suggesting that their integration could provide complementary insights into vocal function (Deliyski and Hillman, 2010; Mehta et al., 2010; Schlegel et al., 2021). Therefore, the combination of time-synchronized acoustic and HSV recordings holds considerable potential to enhance voice analysis.

Apart from our work in Schraut et al. (2025), previous studies have primarily focused on distinguishing between normal and pathological voices. However, the severity of voice disorders and associated characteristics (e.g., hoarseness) is typically continuous in nature. While auditory-perceptual assessments attempt to capture this continuum through coarse grading scales, a more fine-grained evaluation could be achieved through quantitative analysis, allowing for tracking subtle changes in vocal function over time.

Integrating ML-based analysis of HSV and acoustic recordings into clinical workflows will enhance the objectivity, consistency, and efficiency of voice assessments. By providing quantitative and reproducible estimates of hoarseness severity, such tools can support clinical decision making, facilitate early detection of functional voice impairments, and enable detailed monitoring of treatment outcomes. This would reduce dependence on subjective ratings, help standardize diagnostic procedures across institutions, and ultimately improve the quality of care for patients with voice disorders.

This study investigates a ML-based approach for assessing hoarseness severity in functional dysphonia based on synchronous HSV and acoustic recordings. Specifically, a classification model is developed using quantitative parameters extracted from HSV recordings. This includes identifying an appropriate classification algorithm and determining a minimal-optimal subset of features.

Subsequently, the proposed videoendoscopic model will be combined with the acoustic model previously developed in Schraut et al. (2025) to form an ensemble model based on time-synchronized HSV and acoustic recordings. The models will be evaluated based on their ability to quantify hoarseness severity H and detect relative changes in severity over time.

In addition to the HSV-based ensemble model, this study will explore an ensemble model that integrates data from all commonly performed voice examinations. For this purpose, the results from Schlegel et al. (2020a) will be considered, where questionnaires and acoustic/aerodynamic parameters commonly acquired in clinical practice were reduced to a relevant subset of four parameters.

## 2 Materials and methods

### 2.1 Database

The data used in this study were obtained from patient consultations and studies conducted at the Division of Phoniatrics and Pediatric Audiology at the University Hospital Erlangen. All studies were approved by the ethics committee at the Faculty of Medicine at Friedrich-Alexander-Universität Erlangen-Nürnberg (reference numbers 290_13 B, 61_18 B, 219_19 B and 139_20 B). All methods were carried out in accordance with relevant guidelines and regulations. Written informed consent was obtained by the subjects.

Three databases comprising different modalities and examinations are used in this study: a database of HSV recordings ( $D_{V}$ ), a database of HSV-synchronized acoustic recordings ( $D_{A}$ ), and a database of clinical parameters obtained in separate functional voice assessments ( $D_{C}$ ). In this section, the acquisition and pre-processing of these data are described.

#### 2.1.1 Data acquisition

In the HSV examination, subjects were instructed to phonate the sustained vowel /i/ at a habitual pitch and loudness level, while the rigid endoscope was positioned in their oral cavity and held slightly above the vocal folds. During sustained phonation, a high-speed video recording of the vocal fold movement and a synchronous acoustic recording were captured.

The HSV recordings were acquired using two imaging setups: (A) the *KayPENTAX* system (camera: *Photron FASTCAM MC2*; light source: *Model 7152B Xenon;* endoscope: 70°, rigid; frame rate: 4000 fps; resolution: 512 × 256 pixel) from *PENTAX Medical* (Montvale, NJ) and (B) the *OpenHSV* system (camera: *IDT CCM-1540*; light source: *Karl Storz Power LED 300*; endoscope: 70°, rigid; frame rate: 4000 fps; resolution: 1024 × 1,024 pixel) by Kist et al. (2021a). Both systems include a clip microphone, which was located near the base of the camera with a distance of approximately 30 cm from the subject’s mouth. The *KayPENTAX* system employs the *Audio Technica ASP-0091* (*PENTAX* model #7175–6,000) lavalier microphone with a sampling rate of 40 kHz, while the *OpenHSV* system uses the *DPA 4060* lavalier microphone with a sampling rate of 80 kHz (Kist et al., 2021a). The acoustic recordings of both HSV systems were down-sampled to 22.05 kHz, which was found to be sufficient for voice quality assessment (Schraut et al., 2025).

The clinical parameters defined by Schlegel et al. (2020a) were acquired in a separate phoniatric examination. Here, several voice recordings were captured to determine the voice range profile of the subject, from which the maximum achievable frequency ( $F^{\text{max}}$ ) and intensity ( $I^{\text{max}}$ ) could be derived. Furthermore, a recording of the sustained vowel /a/ at a comfortable pitch and intensity was obtained to determine acoustic jitter percent ( $Jit_{\%}$ ). The recordings were captured and analyzed using the *lingWAVES Voice Diagnostic Center* system, placing the *lingWAVES SPL Meter II* microphone at a distance of 30 cm from the subjects’ mouth. The recorded signals were sampled at 22.05 kHz with a resolution of 16 bit/sample. Finally, the subjects were asked to complete questionnaires regarding the self-assessment of their voice. This includes the relevant VRQOL and/or the VHI.

In addition to the HSV and phoniatric examinations, a recording of continuous speech (*Der Nordwind und die Sonne* (Schneider-Stickler and Bigenzahn, 2013)) was obtained from each subject using the phoniatric setting. Subsequently, this recording was evaluated auditory-perceptively by an expert according to the RBH scale, resulting in a corresponding RBH rating for each patient visit. If patients underwent both the HSV and voice therapy examination during a single visit, the resulting recordings and parameters share the same RBH score. This overlap is taken into consideration when dividing the data as explained in Section 2.1.4.

Overall, 1,641 visits from 1,110 subjects (725 females, 385 males) were considered for this study. Visits were removed from the respective HSV database if the quality of the underlying recording was found to be insufficient. Likewise, visits were excluded if not all clinical parameters were available, with an exception of VRQOL (see Section 2.1.3.3).

In total, the HSV database ( $D_{V}$ ) includes 457 recordings from 377 subjects (242 females, 135 males). Subjects consist of 193 healthy controls and 184 patients with voice impairment. The HSV-based acoustic database ( $D_{A}$ ) contains 634 recordings from 505 subjects (321 females, 184 males). Subjects included 288 healthy controls and 266 patients with voice impairment. The clinical database ( $D_{C}$ ) includes 923 examinations from 729 subjects (453 females, 276 males). Here, subjects comprise 400 healthy controls and 329 patients with voice impairment.

All voice disorders within databases $D_{V}$ and $D_{C}$ are caused by functional dysphonia. However, within database $D_{A}$ , 109 of the 266 voice impairments are caused by different voice disorders such as vocal fold polyps, nodules, recurrent paresis, laryngitis, vocal insufficiency, atrophy, Reinke’s edema, etc. These were added to account for more recordings with increased hoarseness, as auditory assessment, unlike visual assessment, can be performed independently of the underlying voice disorder.

#### 2.1.2 Target labels

A supervised classification approach was chosen to derive a continuous hoarseness severity score from voice parameters. Each recording in $D_{V}$ , $D_{A}$ , and $D_{C}$ was assigned a target label based on the clinical RBH rating, using the overall hoarseness score $H\in \left\{0,\;1,\;2,\;3\right\}$ .

Since this is a retrospective study, most recordings were obtained from routine clinical practice, where assessments were conducted by a single voice therapist. These evaluations were primarily based on continuous speech samples. Given the inherent inter- and intra-rater variability in subjective voice assessments as well as potential differences in voice characteristics between continuous speech and sustained phonation, a perfect alignment between ratings and recordings cannot be assumed (Lu and Matteson, 2014).

To account for this, recordings were grouped into two hoarseness levels: H<2 (normal / mild hoarseness) and H≥2 (moderate / severe hoarseness), giving classification models some leeway for minor adjustments. While this frames the task as binary classification, the estimated posterior probability $\hat{y}\in \left[0,\;1\right]$ is treated as a continuous (i.e., interval-scaled) measure of hoarseness severity, where $\hat{y}=0$ corresponds to a normal voice and $\hat{y}=1$ represents severe hoarseness.

#### 2.1.3 Feature extraction

All HSV video and audio recordings were cut to a duration of 250 ms (i.e., 1,000 frames) of sustained phonation, which meets the minimum requirement of 20 phonation cycles for the analysis of HSV data (Schlegel et al., 2018). This restriction of the signal length was necessary because many subjects, in particular patients with voice disorders, were not able to sustain phonation for a longer period of time.

##### 2.1.3.1 Glottal parameters (HSV)

**Figure 1:**
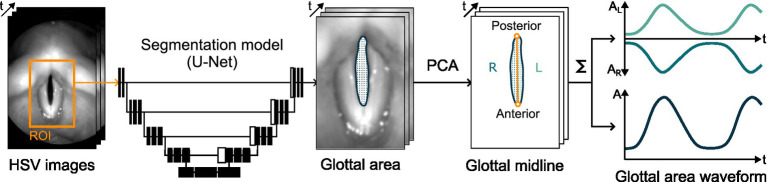
Illustration of the processing of HSV image data into total and partial GAWs using DL-based glottis segmentation and PCA-based midline detection.

The extraction of glottal parameters from high-speed endoscopic videos was based on total and partial GAWs. Figure 1 illustrates the process of obtaining these signals. For each video frame, a DL model automatically identifies the glottal area between the vocal folds. Specifically, a convolutional neural network architecture based on U-Net segments the image to determine which pixels belong to the glottal area (Döllinger et al., 2022; Gómez et al., 2020). Principal component analysis (PCA) is then applied to define a midline that bisects this area, assigning it to the left and right vocal folds (Kist et al., 2021b). Finally, summing the partial and total glottal areas per frame yields the left, right, and total GAWs, which serve as proxies for vocal fold motion.

Table 1 summarizes the parameters extracted from the GAWs. These parameters were computed based on either the full signal, windowed analysis, or phonation cycles. Phonation cycles were determined for each recording through fundamental frequency analysis (Kist et al., 2021b).

**Table 1:** Summary of the 48 glottal features extracted from the GAW.

Parameter	Abbreviation	Unit	Statistics	Source
Fundamental frequency measures				
Fundamental frequency	F_0_	Hz	mean, std.	
Perturbation measures				
Mean jitter	mJit	s	-	Horii (1980)
Period variability index	PVI	a.u.	-	Deal and Emanuel (1978)
**Time periodicity**	**TP**	a.u.	mean, **std.**	Qiu et al. (2003)
Mean shimmer	mShim	a.u.	-	Horii (1980)
**Amplitude variability index**	**AVI**	a.u.	-	Deal and Emanuel (1978)
Amplitude periodicity	AP	a.u.	mean, std.	Qiu et al. (2003)
Energy perturbation factor	EPF	a.u.	-	Kasuya et al. (1993)
Glottal dynamic characteristics				
**Closing quotient**	**CQ**	a.u.	mean, **std.**	Holmberg et al. (1988)
Open quotient	OQ	a.u.	mean, std.	Timcke et al. (1958)
Plateau quotient	PQ	a.u.	mean, std.	Mehta et al. (2011)
Speed quotient	SQ	a.u.	mean, std.	Timcke et al. (1958)
**Glottal area index**	**GAI**	a.u.	**mean**, std.	Chen et al. (2013)
**Glottal gap index**	**GGI**	a.u.	mean, **std.**	Kunduk et al. (2010)
Mechanical measures				
Amplitude-length ratio	ALR	a.u.	mean, std.	Titze (2000)
Stiffness	STF	a.u.	mean, std.	Munhall et al. (1985)
Amplitude quotient	AQ	a.u.	mean, std.	Schlegel et al. (2019)
Symmetry measures				
Phase asymmetry	PA	a.u.	mean, std.	de Jesus Goncalves (2015)
**Phase asymmetry index**	**PAI**	a.u.	mean, **std.**	de Jesus Goncalves (2015)
Noise measures				
Cepstral peak magnitude	CPM	dB	-	Kasuya et al. (1993)
Smoothed cepstral peak prominence	CPPS	dB	-	Kasuya et al. (1993)
**Harmonics intensity**	**HI**	a.u.	-	Hiraoka et al. (1984)
Harmonics-to-noise ratio	HNR	dB	-	Yumoto et al. (1982)
Waveform matching coefficient	WMC	a.u.	mean, max.	Lessing (2007)
**Normalized noise energy**	**NNE**	dB	mean, **std.**	Kasuya et al. (1986)
Signal-to-noise ratio	SNR	dB	mean, std.	Klingholz (1987)
Spectral flatness	SF	a.u.	-	Lessing (2007)
Nyquist plot measures				
Trajectory consistency	TC	a.u.	mean, std.	Arias-Vergara et al. (2023)
**Within trajectory variability**	**WTV**	a.u.	**mean**, **std.**	Arias-Vergara et al. (2023)

The 10 most relevant features as determined by the relevance score defined in Section 2.2.2 are highlighted in bold type.

A total of 48 features were considered based on previous work, capturing various aspects of vocal fold behavior (Arias-Vergara et al., 2023; Schlegel, 2020; Schlegel et al., 2020b). Perturbation measures included cycle-to-cycle variability in period, amplitude, and signal energy (e.g., mJit , mShim ), quantifying the periodicity of vocal fold oscillations associated with vocal roughness (Horii, 1980). Glottal dynamics were characterized by parameters such as OQ (proportion of each cycle the glottis remains open), CQ (duration of the glottis closing phase), SQ (ratio between opening and closing durations), and the GGI (degree of incomplete closure), which relates to breathiness and vocal efficiency (Holmberg et al., 1988; Kunduk et al., 2010; Timcke et al., 1958). Mechanical features included ALR and AQ , reflecting the extent and velocity of vocal fold deflections (Schlegel et al., 2019; Titze, 2000). Symmetry measures such as the PAI , which quantifies the synchronicity of vocal fold motion by measuring the phase shift between left and right fold oscillations, have been associated with a rough voice (de Jesus Goncalves, 2015). Noise measures, including HI , NNE , and HNR , assessed the proportion of periodic versus aperiodic components in the signal and are relevant to perceived roughness and breathiness (Hiraoka et al., 1984; Kasuya et al., 1986; Yumoto et al., 1982). Nyquist plot-based features, like WTV , analyzed the overall consistency of glottal cycles using amplitude-phase representations of the GAW (Arias-Vergara et al., 2023). Parameters highly dependent on the camera angle, such as glottal spatial symmetry measures and those derived from the phonovibrogram (PVG), were excluded from the analysis based on the findings in Veltrup et al. (2023). Additionally, redundant or ill-designed parameters were omitted (Schlegel et al., 2020b; Schlegel et al., 2019).

All processing steps described were carried out using our publicly available software, *Glottis Analysis Tools* (GAT) (Kist et al., 2021b).

##### 2.1.3.2 Acoustic parameters (HSV)

Based on our previous work in Schraut et al. (2024) and Schraut et al. (2025), the following parameters were extracted from the acoustic signal recorded during HSV: mean smoothed cepstral peak prominence ( $CPPS^{\mathrm{mean}}$ ), harmonics intensity ( HI ), std. spectral centroid ( $S_{\mathrm{centroid}}^{std}$ ), mean jitter ( mJit ), and mean peak-to-peak amplitude ( $A^{\mathrm{mean}}$ ). These features quantify the harmonicity, frequency variability, and intensity of the acoustic signal.

##### 2.1.3.3 Clinical parameters

The clinical parameters include the final set determined in Schlegel et al. (2020a): VRQOL , $F^{\text{max}}$ , $I^{\text{max}}$ , $Jit_{\%}$ . For 226 visits, the VRQOL was not available, but the VHI was recorded instead. Based on 518 visits, where both the VRQOL and VHI were available, a Pearson correlation of −0.949 was found between the two questionnaires, reflecting the results of related studies (Portone et al., 2007). Consequently, where applicable, the VRQOL was imputed using linear regression on the VHI .

#### 2.1.4 Data split

The databases were split into training and hold-out test sets to guarantee an unbiased evaluation of the classification models. This was done separately for the development of the video model (Section 2.2) and the evaluation of the multi-sensor approach (Section 2.3), as the latter required an overlap of visits from all three databases. The test data was selected as follows.

First, all visits originating from the same patient taken at different points in time were reserved for the test set. These visits will be used for evaluating the models’ capability to quantify relative change in hoarseness. Next, further visits were randomly selected in order to balance out the resulting hoarseness distribution as evenly as possible. As there were too few visits with hoarseness level H=3 to achieve a uniform distribution (without impacting model training), missing visits were filled with hoarseness level H=2 to achieve an even distribution for binary classification. It was ensured that there was no overlap of subjects between the training and test set.

**Figure 2:**
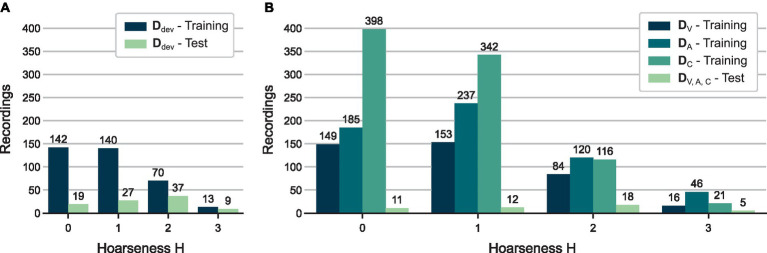
Distributions of auditory-perceptual hoarseness ratings H for all training and test sets used in **(A)** HSV model development (see Section 2.2) and **(B)** evaluation of multi-sensor approaches (see Section 2.3).

For model development (Section 2.2), the database $D_{V}$ was split into 365 training visits ( $D_{dev}^{\mathrm{train}}$ ) and 92 test visits ( $D_{dev}^{\mathrm{test}}$ ), representing approximately 20% of $D_{V}$ . Figure 2A shows the hoarseness distributions of the resulting training and test set.

For evaluation of the multi-sensor approach (Section 2.3), the previously determined test set was reduced to visits recorded in all three databases. Consequently, 46 visits were reserved for the test set $D_{V}^{\mathrm{test}}=D_{A}^{\mathrm{test}}=D_{C}^{\mathrm{test}}$ . The remaining visits were used for model training, resulting in 402 visits in $D_{V}^{\mathrm{train}}$ , 588 visits in $D_{A}^{\mathrm{train}}$ , and 877 visits in $D_{C}^{\mathrm{train}}$ . Figure 2B shows the hoarseness distributions of the resulting training and test sets.

The age and sex distributions of each respective training and test set are available in Supplementary Figures 1, 2.

### 2.2 HSV model development

This section describes the development of a classification model for hoarseness severity estimation based on high-speed endoscopic video data. The model and parameter selection methodology builds upon our previous work on hoarseness classification using HSV audio data (Schraut et al., 2025). Here, $D_{dev}^{\mathrm{train}}$ and $D_{dev}^{\mathrm{test}}$ were used as training and test datasets (see Figure 2A).

#### 2.2.1 Model selection

A model selection was performed to identify suitable classification algorithms for hoarseness severity estimation based on HSV data. Since hoarseness severity is treated as a continuous variable, only classification models that provide a probabilistic output were considered. Based on prior research and best practices for classification of tabular data, a range of linear, non-linear, ensemble- and neural network-based classification algorithms were evaluated (Schraut et al., 2025; Schraut et al., 2024). These included logistic regression (LR) (Hosmer et al., 2013), support vector machines (SVM) with linear and radial basis function (RBF) kernels (Cristianini and Shawe-Taylor, 2000), decision tree (DT) (Rokach and Maimon, 2005), adaptive boosting (AdaBoost) (Schapire, 2013), LogitBoost (Friedman et al., 2000), extreme gradient boosting (XGBoost) (Chen and Guestrin, 2016), light gradient boosting machine (LGBM) (Ke et al., 2017), category boosting (CatBoost) (Prokhorenkova et al., 2018), and a deep tabular data learning architecture (TabNet) (Arik and Pfister, 2021).

For each model, a five-fold cross-validation (CV) was performed on the training set. In each fold, 80% of the training data was allocated for training, while the remaining 20% served as validation set. Hyperparameter selection was conducted exclusively on the training data, i.e., excluding the respective validation set of each fold. Here, an exhaustive grid search was conducted based on a separate three-fold CV on the training data, using a pre-defined hyperparameter grid for each classification model (see Supplementary Table 1). Hyperparameters were selected so that the mean logarithmic loss was minimized. Feature standardization was performed using the mean and standard deviation of the training data. To address the class imbalance between H<2 and H≥2 , class weights were assigned inversely proportional to class frequencies. Model performance was assessed on the validation sets using accuracy, sensitivity, and specificity as evaluation metrics.

#### 2.2.2 Feature selection

In this study, feature selection was performed to identify a minimal-optimal subset of HSV parameters. Our approach aimed to reduce the number of features while maintaining sufficient model performance, prioritizing the clinical relevance and interpretability of selected parameters over purely maximizing predictive accuracy.

Non-parametric statistical methods were applied to all features, as the Shapiro–Wilk test indicated that most features did not follow a normal distribution across all hoarseness levels (Shapiro and Wilk, 1965).

First, the Kruskal-Wallis test was used to assess all parameters for significant differences across hoarseness levels H . Parameters that did not show significant differences between any hoarseness levels ( p>0.05 ) were excluded from the feature set (McKight and Najab, 2010).

The remaining features were ranked by relevance using a combination of four feature selection methods. In addition to the Kruskal-Wallis test, the ReliefF algorithm was used to assess the ability of features to distinguish between instances with similar and dissimilar hoarseness levels (Urbanowicz et al., 2018). Spearman’s rank correlation coefficient ρ was calculated to evaluate the strength of the monotonic relationship between each feature and hoarseness H (Zar, 2005). Mutual information was also considered to capture non-linear relationships (Kraskov et al., 2004). The final relevance score of each feature was derived by averaging the normalized scores from these methods. By incorporating multiple measures of class separability and statistical dependence, this approach enhances robustness of results against the limitations of the individual methods (Gómez-García et al., 2019).

Subsequently, Spearman’s rank correlation coefficient was computed between features to eliminate redundancy. Features with a strong correlation |ρ|≥0.9 were grouped, and the feature with the highest relevance score in each group was retained, while the others were discarded (Ding and Peng, 2005).

Afterwards, the remaining feature set was reduced to the 10 most important features based on their previously determined relevance score. A five-fold CV was performed to ensure these features adequately capture the hoarseness-related information of the full feature set.

Finally, the feature set was fine-tuned using the embedded methods of the previously selected classification models. Specifically, the remaining features were ranked using the embedded feature importance scores, i.e., model coefficients (LR) or information gain-based feature importance values (XGBoost) (Chen and Guestrin, 2016; Guyon and Elisseeff, 2003; Jovic et al., 2015). Going from highest to lowest ranked feature, a greedy forward selection was performed for each classification model. At each iteration, the feature subset was evaluated in a five-fold CV. A feature set was selected, if the addition of the subsequent feature did not lead to an increase in the model’s objective function (i.e., negative logarithmic loss).

The resulting model and feature set combinations were then compared based on the results of the five-fold CV to determine a final model.

#### 2.2.3 Model evaluation

The selected classification model and feature set were trained on the complete training set ( $D_{dev}^{\mathrm{train}}$ ) and evaluated on the hold-out test set ( $D_{dev}^{\mathrm{test}}$ ). In addition to classification metrics, a more detailed analysis of the final approach was conducted.

First, the model’s ability to quantitatively represent hoarseness severity was examined by analyzing the correlation between predicted probability scores $\hat{y}$ and subjectively determined hoarseness ratings H for the test set.

Second, the model’s ability to capture relative changes in hoarseness severity (i.e., improvement, worsening, or no change) was evaluated. For 31 test subjects with multiple recordings taken at different time points (e.g., before and after voice therapy), the observed differences in hoarseness severity $\Delta H=H_{\mathrm{post}}-H_{pre}$ were compared to the corresponding differences in predicted scores $\Delta \hat{y}={\hat{y}}_{\mathrm{post}}-{\hat{y}}_{pre}$ in terms of quantitative agreement.

### 2.3 Multi-sensor approach

This section describes the evaluation of the final models based on videoendoscopic data ( $m_{V}$ ), HSV-based acoustic data ( $m_{A}$ ), and additional clinical parameters ( $m_{C}$ ). Subsequently, two ensemble methods were constructed by combining the pre-trained HSV-based models ( $m_{VA}$ ) and all models ( $m_{VAC}$ ), respectively.

#### 2.3.1 Evaluation of individual models

The evaluation included the HSV model $m_{V}$ developed in Section 2.2, i.e., XGBoost and feature set $X_{V}=\left\{WTV^{\mathrm{mean}},\;NNE^{std},\;HI,\;GGI^{std},\;CQ^{std}\right\}\text{.}$ The HSV-based audio model $m_{A}$ defined in Schraut et al. (2025) uses LR and the acoustic features $X_{A}=\left\{CPPS^{\mathrm{mean}},\;HI,\;\mathrm{mJit},\;S_{\mathrm{centroid}}^{std},\;A^{\mathrm{mean}}\right\}$ . The clinical parameter model $m_{C}$ is based on the feature set $X_{C}=\left\{\mathrm{VRQOL},\;F^{\text{max}},\;I^{\text{max}},\;Jit_{\%}\right\}$ defined in Schlegel et al. (2020b) and uses LR as classification algorithm, as determined in a model selection on the training set $D_{C}^{\mathrm{train}}$ . The complete results of the model selection are provided in Supplementary Table 2.

Each model was evaluated using five-fold CV on the training sets defined in Figure 2B. Classification performance was assessed analogous to Section 2.2.2, considering accuracy, sensitivity, and specificity. In addition, the receiver operating characteristic (ROC) curve and the corresponding area under the curve (AUC) score were used for the evaluating the final models. Furthermore, the correlation between predicted probability scores $\hat{y}$ and subjective hoarseness ratings H was analyzed based on out-of-sample predictions from each validation set, providing comparable results to the relatively small hold-out test set.

Following CV, the models were trained on their respective full training sets and evaluated on the shared hold-out test set. The analysis of the test results includes classification metrics, the ROC curve, correlation between $\hat{y}$ and H , and correlation between $\Delta \hat{y}$ and ΔH . The latter was evaluated using 20 pre- and post-recordings from 14 test subjects. Due to the relatively small size of the test set, bootstrapping (10,000 resamples) was performed to estimate 95% confidence intervals for the classification metrics, providing a more robust comparison of model performance on the test set.

In addition, the correlation between the parameters of the feature sets $X_{V}$ , $X_{A}$ , and $X_{C}$ and hoarseness H was examined based on the respective complete databases $D_{V}$ , $D_{A}$ , and $D_{C}$ .

#### 2.3.2 Evaluation of ensemble models

Two ensemble models were investigated. The first, $m_{VA}=\left\{m_{V},\;m_{A}\right\}$ , combines the models $m_{V}$ and $m_{A}$ based on time-synchronous videoendoscopic and acoustic data. The second, $m_{VAC}=\left\{m_{V},\;m_{A},\;m_{C}\right\}$ , extends this approach by incorporating the clinical parameter model $m_{C}$ .

The ensemble models were constructed without additional model training. Instead, the individual models from Section 2.3.1 were used as-is, having been trained on their respective full training sets (see Figure 2B). Ensemble predictions were obtained by averaging the predictions of the individual models with equal weights. Alternative weighting schemes based on the classification performance of individual models in the five-fold cross-validation were investigated, but did not yield performance improvements.

Analogous to Section 2.3.1, all ensemble models were evaluated on the hold-out test set shared between the databases.

## 3 Results

### 3.1 HSV model development

#### 3.1.1 Model selection

Table 2 shows the results of the five-fold CV for all classification models. Some models, particularly DT, LogitBoost, and CatBoost, tend to underestimate hoarseness severity, as indicated by low sensitivity and high specificity. XGBoost achieves a slight advantage over the remaining ensemble-based methods (AdaBoost, LGBM), while also outperforming TabNet. LR and SVM models show a similar performance to XGBoost, with LR providing a notable balance between sensitivity and specificity.

**Table 2:** Classification results of the model selection using all features defined in Table 1.

Model	Accuracy	Sensitivity	Specificity
**LR**	**0.729 ± 0.062**	**0.638 ± 0.052**	**0.756 ± 0.074**
SVM (linear)	0.781 ± 0.040	0.578 ± 0.062	0.841 ± 0.056
SVM (RBF)	0.781 ± 0.056	0.566 ± 0.065	0.844 ± 0.079
DT	0.767 ± 0.066	0.387 ± 0.064	0.879 ± 0.082
AdaBoost	0.753 ± 0.057	0.542 ± 0.068	0.816 ± 0.090
LogitBoost	0.805 ± 0.006	0.385 ± 0.101	0.929 ± 0.028
LGBM	0.751 ± 0.035	0.530 ± 0.086	0.816 ± 0.039
**XGBoost**	**0.751 ± 0.059**	**0.565 ± 0.092**	**0.805 ± 0.072**
CatBoost	0.822 ± 0.031	0.435 ± 0.117	0.936 ± 0.042
TabNet	0.701 ± 0.037	0.517 ± 0.163	0.755 ± 0.070

For each model, results are reported in terms of mean and standard deviation of the 5-fold CV. Selected models are highlighted in bold type.

Based on these results, LR and XGBoost were selected for subsequent feature selection, representing both a linear and ensemble-based model architecture.

#### 3.1.2 Feature selection

The Kruskal-Wallis test showed significant differences between hoarseness levels for 32 of the 48 extracted features. Features that did not show a significant difference were discarded. Next, using Spearman’s rank correlation coefficient, five very strongly correlated features were identified and removed, leaving 27 nonredundant features. The 10 most relevant features determined from the remaining subset by the relevance score described in Section 2.2.2 are marked in Table 1.

Table 3 summarizes the results of the five-fold CV using the full feature set, the 10 most relevant features, and the final feature sets determined by embedded methods for LR and XGBoost (see Section 2.2.2). For both models, classification accuracy was maintained throughout feature selection, while a better balance between sensitivity and specificity was achieved. Overall, there is no notable difference in the models’ performance.

**Table 3:** Classification results using the full feature set, the 10 most relevant features, and the final feature sets determined by embedded methods.

Set	LR			XGBoost		
	Accuracy	Sensitivity	Specificity	Accuracy	Sensitivity	Specificity
Full	0.729 ± 0.062	0.638 ± 0.052	0.756 ± 0.074	0.751 ± 0.059	0.565 ± 0.092	0.805 ± 0.072
Top 10 (Filter)	0.710 ± 0.059	0.649 ± 0.090	0.727 ± 0.069	0.764 ± 0.067	0.588 ± 0.117	0.816 ± 0.061
Final (Embedded)	0.737 ± 0.055	0.649 ± 0.090	0.763 ± 0.062	**0.751 ± 0.037**	**0.636 ± 0.137**	**0.784 ± 0.047**

For each model and set, results are reported in terms of mean and standard deviation of the 5-fold CV. The results of the selected model and feature set combination are highlighted in bold type.

Due to the slightly better results and more consistent performance in the selection process, XGBoost and feature set $X_{V}=\left\{WTV^{\mathrm{mean}},\;NNE^{std},\;HI,GGI^{std},\;CQ^{std}\right\}$ were chosen as the final model $m_{V}$ .

#### 3.1.3 Model evaluation

The final model, XGBoost and $X_{V}$ , was trained on the complete training set $D_{dev}^{\mathrm{train}}$ and evaluated on the hold-out test set $D_{dev}^{\mathrm{test}}$ . An accuracy of 0.663, a sensitivity of 0.652 and a specificity of 0.674 were achieved on the test set, showing a slight decrease in performance compared to model validation (see Table 3).

**Figure 3:**
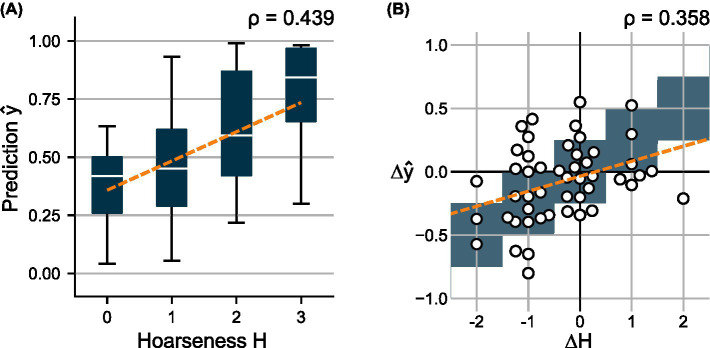
**(A)** Distributions of predicted scores $\hat{y}$ over the subjectively determined hoarseness levels H for test set $D_{dev}^{\mathrm{test}}$ . **(B)** Change in predicted scores $\Delta \hat{y}$ over the change in hoarseness levels ΔH for 49 pre- and post- recording pairs. A regression line was fitted to indicate the relationship between prediction and ground truth, respectively.

Figure 3A shows the distributions of predicted probability scores $\hat{y}$ over the auditory-perceptual hoarseness ratings H for the test set. As indicated by the fitted regression line, the distributions show a clear positive trend between predictions and subjective ratings. Overall, the model achieves a moderate correlation of 0.439 between $\hat{y}$ and H .

Using 49 pre- and post-recording pairs, Figure 3B compares the difference in subjective ratings ΔH to the change in predicted scores $\Delta \hat{y}$ . Here, a weak correlation of 0.358 is obtained between $\Delta \hat{y}$ and ΔH .

### 3.2 Multi-sensor approach

#### 3.2.1 Evaluation of individual models

The videoendoscopic model $m_{V}$ , acoustic model $m_{A}$ , and clinical model $m_{C}$ were first evaluated in a five-fold CV using the training sets $D_{V}^{\mathrm{train}}$ , $D_{A}^{\mathrm{train}}$ , and $D_{C}^{\mathrm{train}}$ . Subsequently, all models were trained using the complete training sets and evaluated on the shared hold-out test set. The hyperparameters of each final model can be found in Supplementary Table 1.

**Figure 4:**
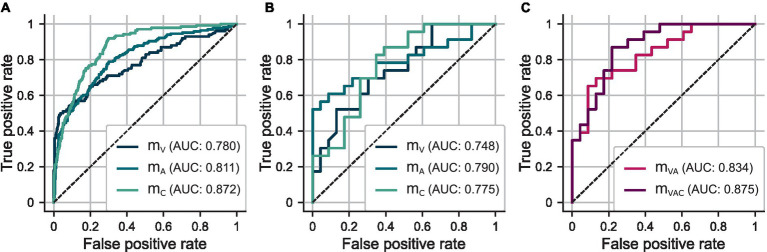
**(A)** ROC curves for out-of-sample predictions determined in five-fold CV using $m_{V}$ and $D_{V}^{\mathrm{train}}$ , $m_{A}$ and $D_{A}^{\mathrm{train}}$ , and $m_{C}$ and $D_{C}^{\mathrm{train}}$ . **(B)** ROC curves for test set predictions using $m_{V}$ , $m_{A}$ , and $m_{C}$ . **(C)** ROC curves for test set predictions using ensemble models $m_{VA}$ and $m_{VAC}$ .

**Figure 5:**
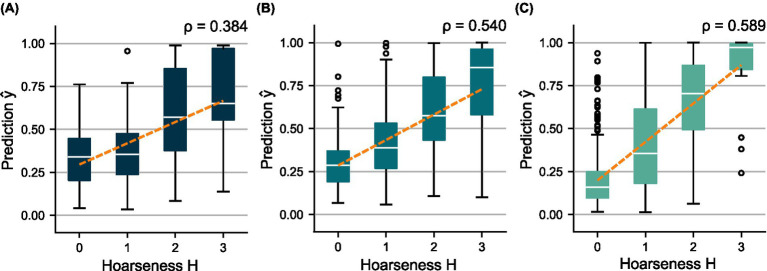
Distributions of the out-of-sample predictions $\hat{y}$ over the subjectively determined hoarseness levels H using **(A)** $m_{V}$ and $D_{V}^{\mathrm{train}}$ , **(B)** $m_{A}$ and $D_{A}^{\mathrm{train}}$ , and **(C)** $m_{C}$ and $D_{C}^{\mathrm{train}}$ . A regression line was fitted to indicate the relationship between prediction and ground truth, respectively.

Table 4 summarizes the results of the five-fold CV in terms of accuracy, sensitivity, and specificity. The ROC curve of each model is depicted in Figure 4A. Figure 5 shows the distributions of predicted scores $\hat{y}$ over the subjective hoarseness ratings H for the training sets. As mentioned in Section 2.3.1, the predicted scores represent the out-of-sample predictions for each validation split of the fivefold CV. The corresponding confusion matrices of all models can be found in Supplementary Figure 3.

**Table 4:** Classification results using the respective model, feature set, and training set of each modality.

Model	Training set	Accuracy	Sensitivity	Specificity
$m_{V}$	$D_{V}^{\mathrm{train}}$	0.764 ± 0.038	0.600 ± 0.117	0.818 ± 0.072
$m_{A}$	$D_{A}^{\mathrm{train}}$	0.757 ± 0.033	0.645 ± 0.101	0.801 ± 0.040
$m_{C}$	$D_{C}^{\mathrm{train}}$	0.802 ± 0.031	0.761 ± 0.112	0.809 ± 0.036

Results are reported in terms of mean and standard deviation of the 5-fold CV.

The HSV-based models, $m_{V}$ and $m_{A}$ , achieve comparable performance regarding classification metrics. However, model $m_{A}$ shows more distinct differences between the prediction distributions for the hoarseness levels. This is reflected in particular by an increase in the correlation between $\hat{y}$ and H from $m_{V}$ ( ρ=0.384 ) to $m_{A}$ ( ρ=0.540 ). Model $m_{C}$ , which is based on additional clinical parameters, clearly outperforms the two HSV-based models in terms of sensitivity, resulting in an increase in both classification accuracy and correlation toward hoarseness ( ρ=0.589 ). These trends are reflected by the ROC curves and the AUC scores achieved on the training sets.

**Figure 6:**
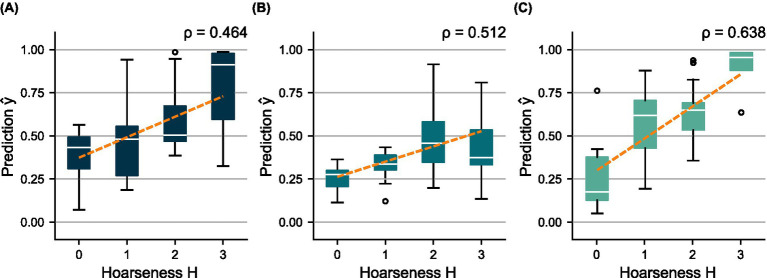
Distributions of predicted scores $\hat{y}$ over the subjectively determined hoarseness levels H for the shared test set using **(A)** $m_{V}$ , **(B)** $m_{A}$ , and **(C)** $m_{C}$ . A regression line was fitted to indicate the relationship between prediction and ground truth, respectively.

Table 5 provides the classification results of $m_{V}$ , $m_{A}$ , and $m_{C}$ on the shared test set. Figure 4B shows the ROC curves of the models. The distributions of predicted test scores $\hat{y}$ over hoarseness levels H are shown in Figure 6. The confusion matrices for the test results can be found in Supplementary Figure 4.

**Table 5:** Classification results on the shared test set using the respective model of each modality as well as the multi-sensor approaches.

Model	Accuracy	Sensitivity	Specificity
$m_{V}$	0.674 (0.543, 0.804)	0.609 (0.400, 0.800)	0.739 (0.550, 0.909)
$m_{A}$	0.717 (0.587, 0.848)	0.435 (0.231, 0.640)	1.000 (1.000, 1.000)
$m_{C}$	0.739 (0.609, 0.870)	0.826 (0.652, 0.960)	0.652 (0.450, 0.840)
$m_{VA}$	0.761 (0.630, 0.870)	0.609 (0.400, 0.808)	0.913 (0.783, 1.000)
$m_{VAC}$	0.783 (0.652, 0.891)	0.783 (0.593, 0.947)	0.783 (0.600, 0.947)

For each model and metric, the 95% confidence intervals determined via bootstrapping are shown in brackets.

While the test results of $m_{V}$ reflect model validation, there is an imbalance between sensitivity and specificity for $m_{A}$ and $m_{C}$ . The prediction distributions show that $m_{A}$ underestimates hoarseness for the test data, especially regarding severe hoarseness H=3 . In turn, $m_{C}$ overestimates hoarseness for mild hoarseness H=1 . However, in both cases, the trend of distributions still indicates greater agreement with hoarseness than $m_{V}$ . This is confirmed by the ROC curves in Figure 4B, as well as the correlation achieved for $m_{A}$ ( ρ=0.512 ) and $m_{C}$ ( ρ=0.638 ) compared to $m_{V}$ ( ρ=0.464 ), which reflects the results of the five-fold CV. Overall, the results suggest a bias due to the small size of the test set.

**Figure 7:**
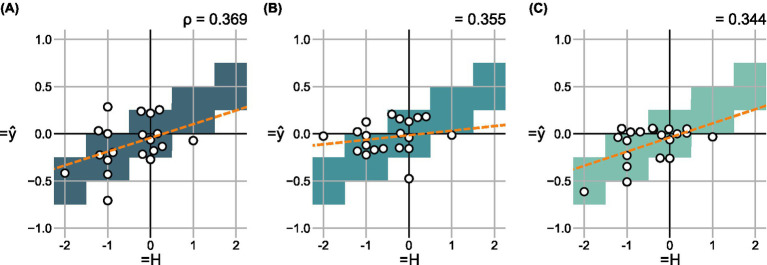
Change in predicted scores $\Delta \hat{y}$ over the change in hoarseness levels ΔH for 20 pre- and post- recording pairs in the shared test set using **(A)** $m_{V}$ , **(B)** $m_{A}$ , and **(C)** $m_{C}$ . For each degree of change, correct value ranges are indicated. A regression line was fitted to indicate the relationship between prediction and ground truth, respectively.

Using 20 pre- and post-recording pairs, Figure 7 compares the difference in scores $\Delta \hat{y}$ predicted by each model to the change in subjective ratings ΔH . There were no particular differences in quality between the models, which all achieve a weak correlation between $\Delta \hat{y}$ and ΔH .

Table 6 summarizes the correlation between $X_{V}$ , $X_{A}$ , and $X_{C}$ and subjective hoarseness ratings H based on the complete databases $D_{V}$ , $D_{A}$ , and $D_{C}$ . Overall, the degree of correlation of the feature sets reflects the resulting performance of the associated models.

**Table 6:** Spearman’s rank correlation coefficient ρ between the final parameters and subjective hoarseness ratings.

Parameter (statistic)	Abbreviation	Spearman’s ρ
Glottal parameters (HSV)		
Within trajectory variability (mean)	$WTV^{\mathrm{mean}}$	0.340
Normalized noise energy (std.)	$NNE^{std}$	0.241
Harmonics intensity	HI	−0.339
Glottal gap index (std.)	$GGI^{std}$	0.322
Closing quotient (std.)	$CQ^{std}$	0.253
Acoustic parameters (HSV)		
Smoothed cepstral peak prominence (mean)	$CPPS^{\mathrm{mean}}$	−0.351
Harmonics intensity	HI	−0.393
Mean jitter	mJit	0.434
Spectral centroid (std.)	$S_{\mathrm{centroid}}^{std}$	0.446
Peak-to-peak amplitude (mean)	$A^{\mathrm{mean}}$	−0.360
Clinical parameters		
Voice related quality of life	VRQOL	−0.541
Maximum achievable frequency	$F^{\text{max}}$	−0.451
Maximum achievable intensity	$I^{\text{max}}$	−0.338
Jitter percent	$Jit_{\%}$	0.336

#### 3.2.2 Evaluation of ensemble models

The models $m_{V}$ , $m_{A}$ , and $m_{C}$ were combined into ensemble models and evaluated on the shared test set. Specifically, the ensemble of the HSV-based models ( $m_{VA}$ ), and the ensemble of all three models ( $m_{VAC}$ ) were investigated.

**Figure 8:**
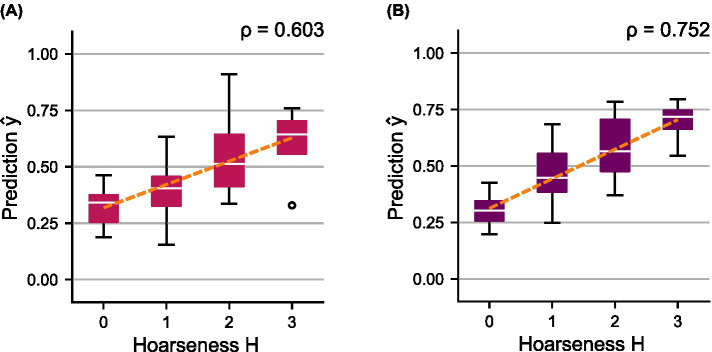
Distributions of predicted scores $\hat{y}$ over the subjectively determined hoarseness levels H for the shared test set using ensemble models **(A)** $m_{VA}$ , and **(B)** $m_{VAC}$ . A regression line was fitted to indicate the relationship between prediction and ground truth, respectively.

Table 5 and Figure 4C compare the classification performance of the ensemble approaches to the individual models on the test set. Figure 8 shows the distributions of predicted test scores $\hat{y}$ over subjective ratings H for the two ensemble models. The confusion matrices of the ensemble models can be found in Supplementary Figure 5.

The combination of the HSV-based models, $m_{VA}$ , achieves a classification performance similar to $m_{C}$ and thus shows a clear improvement compared to the individual models. Ensemble model $m_{VAC}$ achieves the best overall performance, with an ideal balance between sensitivity and specificity. Consequently, the ROC curves as well as the AUC scores show a clear successive increase in performance by combining the models. Compared to their components, both ensemble models show a better trend with regard to the distributions of the predicted probabilities $\hat{y}$ , which is also reflected in a successive increase in correlation toward hoarseness H for $m_{VA}$ ( ρ=0.603 ) and $m_{VAC}$ ( ρ=0.752 ).

**Figure 9:**
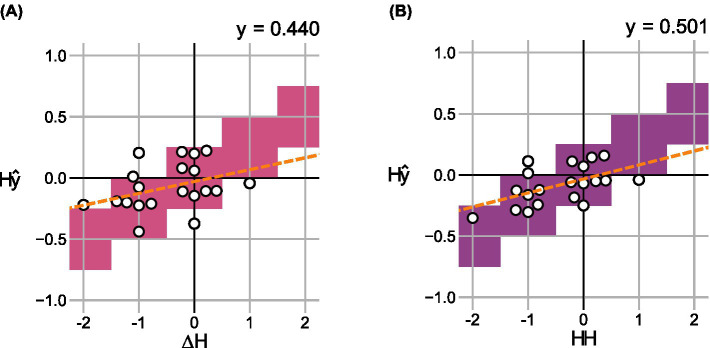
Change in predicted scores $\Delta \hat{y}$ over the change in hoarseness levels ΔH for 20 pre- and post- recording pairs in the shared test set using ensemble models **(A)** $m_{VA}$ , and **(B)** $m_{VAC}$ . For each degree of change, correct value ranges are indicated. A regression line was fitted to indicate the relationship between prediction and ground truth, respectively.

Analogous to Section 3.2.1, Figure 9 compares the difference in scores $\Delta \hat{y}$ to the change in subjective ratings ΔH for both ensemble models. Again, a successive increase in correlation between $\Delta \hat{y}$ and ΔH is achieved for $m_{VA}$ ( ρ=0.440 ) and $m_{VAC}$ ( ρ=0.501 ), outperforming the individual models (see Figure 7).

## 4 Discussion

### 4.1 HSV model development

This study developed a model for estimating hoarseness severity based on glottal parameters extracted from high-speed endoscopic videos. Model development involved the identification of a suitable classification algorithm and the application of filter-, embedded-, and wrapper-based feature selection methods to determine a minimal-optimal feature set.

Notably, the 10 most relevant features identified by filter methods were not confined to a specific subgroup of parameters, but included various characteristics describing perturbation, noise, glottal dynamics, and glottal symmetry (see Table 1). A common aspect of the selected features is their measurement of the irregularity or consistency of the underlying glottal characteristic. While perturbation, noise, and Nyquist plot measures inherently capture irregularity, the standard deviation was identified as a relevant statistic for most glottal dynamics and symmetry parameters.

These consistency-related features were selected for the final feature set $X_{V}=\left\{WTV^{\mathrm{mean}},\;NNE^{std},\;HI,GGI^{std},\;CQ^{std}\right\}$ . $WTV^{\mathrm{mean}}$ holistically captures the consistency of vocal fold movement in both amplitude and phase. HI indicates the proportion of harmonic energy in the signal, which is generally associated with periodic, stable phonation. In contrast, $NNE^{std}$ reflects fluctuations in the noise-to-signal ratio throughout phonation. $CQ^{std}$ describes the consistency in the duration of the vocal folds’ closing phase, and $GGI^{std}$ measures the variability of glottal closure across cycles. Overall, these features provide a complementary characterization of consistency of vocal fold movement, with increased irregularity across these measures generally reflecting greater hoarseness severity.

The parameters identified in this study are consistent with those reported by Arias-Vergara et al. (2023), with six of the ten most relevant features and four of the five features in $X_{V}$ also included in their final feature set. In contrast, there is no overlap with the findings of Schlegel et al. (2020b), whose selected features are predominantly PVG-based, which were not considered in this study.

The selected model, XGBoost and feature set $X_{V}$ , achieved a moderate correlation (0.439) between the predictions $\hat{y}$ and the subjective hoarseness ratings H across 92 test recordings, and a weak correlation (0.358) between the relative changes $\Delta \hat{y}$ and ΔH in 49 pre- and post-recording pairs. The distributions of model predictions $\hat{y}$ and $\Delta \hat{y}$ showed a clear positive trend toward increasing hoarseness, supporting the relationship between glottal irregularities and hoarseness severity (see Figure 3).

### 4.2 Model evaluation

#### 4.2.1 Individual models

In this study, three models were evaluated using data commonly collected in voice examinations. The HSV model, $m_{V}$ (XGBoost, $X_{V}$ ), was based on glottal parameters extracted from high-speed endoscopic video recordings. The acoustic model, $m_{A}$ (LR, $X_{A}$ ), used parameters extracted from HSV-synchronized acoustic recordings. The clinical model, $m_{C}$ (LR, $X_{C}$ ), incorporated a questionnaire score alongside acoustic parameters derived from separate voice samples recorded in a functional voice examination.

These models achieved correlations of 0.384 ( $m_{V}$ ), 0.540 ( $m_{A}$ ), and 0.589 ( $m_{C}$ ) between the out-of-sample predictions $\hat{y}$ and the subjective hoarseness ratings H on their respective training data, with similar correlations of 0.464 ( $m_{V}$ ), 0.512 ( $m_{A}$ ), and 0.638 ( $m_{C}$ ) observed on the shared test set. Both the HSV-based acoustic model $m_{A}$ and the clinical model $m_{C}$ , which relies heavily on acoustic parameters, outperformed the videoendoscopic model $m_{V}$ . Given that the ground truth, hoarseness severity H , is determined through auditory-perceptual assessment, it is expected that models based on the acoustic signal exhibit stronger correlations with the subjective ratings.

While the performance of $m_{V}$ confirms a relationship between glottal function and perceived hoarseness, vocal fold irregularities do not appear to be sufficient to capture hoarseness severity. While HSV sheds light on the mechanical and dynamical behavior of the vocal folds, it does not account for supraglottic influences such as airflow turbulence, resonance, or articulation in the vocal tract, all of which shape the perceived voice quality. This limitation is further reflected in the correlation analyses of individual feature sets, as all reported acoustic features and most clinical features show stronger associations with hoarseness than video-based parameters (see Table 6). Although many glottal and acoustic features capture similar aspects of phonatory irregularity (e.g., signal perturbation and noise), acoustic parameters reflect the perceived output signal, while glottal parameters describe only the source characteristics. This likely explains the stronger correlation of acoustic features with perceptual ratings. The superior performance of $m_{C}$ over $m_{A}$ is likely driven by the subjective self-assessment VRQOL, which shows the strongest correlation with H . Despite being derived from dedicated functional voice recordings, the acoustic parameters in $X_{C}$ show no notable difference in quality compared to those in $X_{A}$ , which were extracted from a single HSV-synchronized recording. On average, parameters in $X_{C}$ and $X_{A}$ display a comparable correlation with H , both of which exceed the correlation observed for glottal parameters in $X_{V}$ . However, it is important to note that the correlations between parameters and hoarseness severity were derived from different databases ( $D_{V}$ , $D_{A}$ , and $D_{C}$ ), thereby limiting the reliability of direct comparisons.

All models showed only weak correlations between the relative change in predictions $\Delta \hat{y}$ and subjective hoarseness ratings ΔH (see Figure 7). However, as these findings are based on only 20 pre- and post-recording pairs, potential bias in the results cannot be ruled out.

#### 4.2.2 Ensemble models

Two ensemble models were evaluated. The first model, $m_{VA}$ , combined the videoendoscopic model $m_{V}$ and the acoustic model $m_{A}$ into an HSV-based ensemble. With a correlation of 0.603 between $\hat{y}$ and H on the shared test set, $m_{VA}$ achieved accuracy comparable to the clinical model $m_{C}$ , representing a notable improvement over the individual HSV-based models. This finding supports previous studies indicating low redundancy between HSV-based video and audio recordings, suggesting that parameters (and consequently models) derived from these modalities can complement each other (Deliyski and Hillman, 2010; Mehta et al., 2010; Schlegel et al., 2021). Additionally, the results demonstrate that objective hoarseness severity estimation comparable to $m_{C}$ , which relies on three dedicated functional voice recordings and a questionnaire, can be achieved using a single HSV examination.

The second ensemble model, $m_{VAC}$ , integrated all three models: $m_{V}$ , $m_{A}$ , and $m_{C}$ . This combination significantly improved classification performance, yielding a strong correlation of 0.752 between $\hat{y}$ and H . The improved accuracy compared to $m_{VA}$ is reasonable, as $X_{C}$ includes voice function-specific parameters that cannot be derived from a single HSV recording. This trend is also observed for the relative change in predictions $\Delta \hat{y}$ and hoarseness ΔH , where both ensemble models achieve only a moderate correlation.

Overall, the results show that a combination of models based on time-synchronized HSV video and audio recordings can achieve a moderate-to-strong correlation with subjective hoarseness ratings. However, current findings suggest that a clinically relevant performance in the objective assessment of functional dysphonia (i.e., hoarseness) cannot yet be realized solely through a single HSV examination and requires further investigation.

A major limiting factor for the predictive accuracy of HSV-based models likely arises from the practical challenges associated with collecting HSV data. During laryngeal examination with a rigid endoscope, the subject’s head and body position must be adjusted according to the endoscope angle to ensure a clear view of the vocal folds. The examiner then anchors the subject’s tongue before the endoscope is inserted into the oral cavity.

However, this procedure can be particularly challenging for patients with severe voice disorders or a pronounced gag reflex, limiting the feasibility of the examination. Many patients are unable to sustain phonation for the required duration, or cannot undergo the examination at all, contributing to the underrepresentation of moderate and severe hoarseness cases (see Figure 2).

Moreover, the examination procedure itself may interfere with the subject’s natural phonation. Studies have reported that rigid endoscopy can influence acoustic parameters, leading to elevated fundamental frequency as well as increased perturbation and noise components (Lim et al., 1998; Ng and Bailey, 2006). As a result, the quality of the recorded sustained vowels is influenced not only by the severity of the voice disorder but also by the subject’s ability to adapt to the procedural requirements (i.e., body and head positioning, tongue placement, rigid endoscope).

These challenging recording conditions hinder the standardized acquisition of HSV data. In the videoendoscopic recordings, this manifests as variations in the distance and angle of the endoscope relative to the vocal folds, camera movements during recording, insufficient lighting or contrast, and occasional obstruction of the glottal opening by surrounding anatomical structures. As demonstrated in previous studies, these inconsistencies can significantly impact subsequent processing steps, such as glottis segmentation and the extraction of glottal parameters (Schlegel et al., 2019; Veltrup et al., 2023).

Additionally, synchronous acoustic recordings often contain noise artifacts from the cooling systems of the HSV camera and light source, as well as from verbal instructions provided by the physician during recording (Schraut et al., 2025). Such noise artifacts negatively affect audio quality, potentially rendering some recordings unsuitable for analysis. These challenges significantly contribute to the limited overlap between HSV and acoustic data in the current databases.

Despite these challenges, HSV has shown great potential in hoarseness severity estimation. Future studies should explore the use of flexible nasal endoscopes, which impose fewer restrictions on natural phonation compared to rigid oral endoscopes, making them potentially more suitable for capturing representative vocal fold behavior (Pietruszewska et al., 2021; Södersten and Lindestad, 1992). In addition, methods to compensate for varying recording conditions (e.g., endoscope distance and angle) should be investigated. This concerns both the post-processing of underlying recordings and the verification of the robustness of glottal parameters.

### 4.3 Comparison to related work

In previous studies by Voigt et al. (2010), Schlegel et al. (2020b), and Arias-Vergara et al. (2023), a maximum accuracy of 0.817, 0.757, and 0.820 was achieved using 12, 13, and 30 features, respectively (see Introduction). However, it is important to note that these studies did not use a hold-out test set. Instead, they reported results based on n-fold CV. Additionally, these studies focused on differentiating normal from pathological voices. In the case of Arias-Vergara et al. (2023), these groups were defined as H=0 and $H\in \left\{2,3\right\}$ . No detailed hoarseness distribution or related information is available for the other studies, making it unclear to what extent samples that are arguably more challenging to classify (i.e., $H\in \left\{1,2\right\}$ ) were represented.

In comparison, for binary classification between H<2 and H≥2 , our videoendoscopic model $m_{V}$ achieved comparable accuracy of 0.764 in five-fold CV and 0.674 on the hold-out test set. Furthermore, by incorporating the acoustic and clinical models to the ensemble, test set accuracy improved successively to 0.761 ( $m_{VA}$ ) and 0.783 ( $m_{VAC}$ ), emphasizing the value of combined time-synchronized HSV and acoustic recordings.

### 4.4 Limitations and future directions

For most patients and visits, only a single expert’s assessment was available. The limitations and adjustments related to the target labels in this study were discussed in Section 2.1.2. Future work should aim to incorporate ratings from multiple experts directly based on the voice recordings. Additionally, averaging multiple subjective ratings allows approaching hoarseness severity estimation as a regression task, which may be better suited for estimating an interval-scaled hoarseness score.

In this study, auditory-perceptual hoarseness ratings served as the ground truth across all modalities. While the relationship between glottal characteristics and hoarseness was confirmed, acoustic-based models are inherently better equipped to capture acoustic characteristics. Therefore, future research should explore the use of a visual ground truth for HSV-based severity estimation models. This could be realized on the basis of the ELS protocol, which includes subjective visual assessments of glottal closure, regularity, and symmetry (Dejonckere et al., 2001).

Based on previous work, this study focused on GAW-based features, while other parameters, such as those derived from the PVG, were excluded due to their sensitivity to recording conditions (Veltrup et al., 2023). As noted in Section 4.2.2, future research should explore post-processing methods to compensate for variable recording conditions and thereby enable the inclusion of additional features. Furthermore, incorporating parameters derived from biomechanical models, such as subglottal pressure, could provide deeper insights into vocal function (Donhauser et al., 2024).

The hold-out test set for evaluating the individual and ensemble models (see Section 2.3) relied on the overlap between the databases of all modalities. Additionally, assessing the relative change in hoarseness required data from multiple visits per patient. Consequently, only a relatively small test set of 46 visits, including 20 pre- and post-recording pairs, was available. This limited sample size can distort the obtained performance metrics, potentially increasing or reducing model accuracy compared to a larger, more diverse dataset. For instance, the acoustic model $m_{A}$ exhibited a noticeable drop in performance between five-fold CV and the test set (see Tables 4, 5). Although the overall trends remained consistent across individual and ensemble models, the small test set reduces the statistical reliability of the results and may not fully reflect actual model capacity. Future work should include validation on larger datasets to derive more robust and clinically meaningful conclusions.

In addition to the limited test set size, the composition of the training data may have influenced classification results. Specifically, there was an overrepresentation of normal voices and mild hoarseness (see Figure 1), as well as a predominance of young adults (ages 18–30) within this subgroup (see Supplementary Figure 1). These imbalances could lead to biased model training, potentially limiting the model’s ability to generalize to older patients or those with more severe impairments. While the test sets used for model evaluation featured a more balanced distribution across hoarseness and age groups, the skewed training data may still affect model robustness. Future work should aim to incorporate more representative samples.

## 5 Conclusion

This study presents a ML-based approach for assessing hoarseness severity dysphonia using synchronous HSV and acoustic recordings, complemented by conventional voice assessments. Our results demonstrate that combining glottal and acoustic parameters from time-synchronized HSV and acoustic recordings offers a more comprehensive evaluation of vocal function, achieving a correlation of 0.603 with auditory-perceptual hoarseness ratings. Further integration of clinical parameters into the ensemble model improved performance, yielding the strongest correlation of 0.752 with subjective hoarseness ratings, underscoring the value of multimodal voice assessment.

Notably, a single HSV examination, when paired with acoustic analysis, can yield a performance comparable to multi-step functional voice assessments. This highlights the potential of HSV to facilitate voice diagnostics by reducing the number of examinations required for objective assessment.

However, practical challenges remain. The use of rigid oral endoscopy can interfere with natural phonation and increase variability in recording conditions (e.g., endoscope positioning), which limits the clinical utility of extracted parameters.

Future work should explore the use of flexible nasal endoscopy to enable more natural phonation and focus on refining glottal parameter extraction to improve model robustness under varying recording conditions. Integrating visual assessment criteria could further enhance objective evaluation based on HSV recordings. Moreover, expanding the available databases, particularly with longitudinal data from patients with functional dysphonia, will be crucial to further improve and validate the proposed models.

## Data Availability

The datasets presented in this article are not readily available because of participant consent restrictions. Requests to access the datasets should be directed to michael.doellinger@uk-erlangen.de.
