# Supplementary material for: Machine learning based assessment of hoarseness severity: a multi-sensor approach centered on high-speed videoendoscopy
Source: Front Artif Intell. 2025 Jun 5;8:1601716. doi: 10.3389/frai.2025.1601716 (PMC12176771; doi:10.3389/frai.2025.1601716)
Supplement: Supplementary file 1 [file Data_Sheet_1.docx]

Supplementary Material

# Data distributions

**
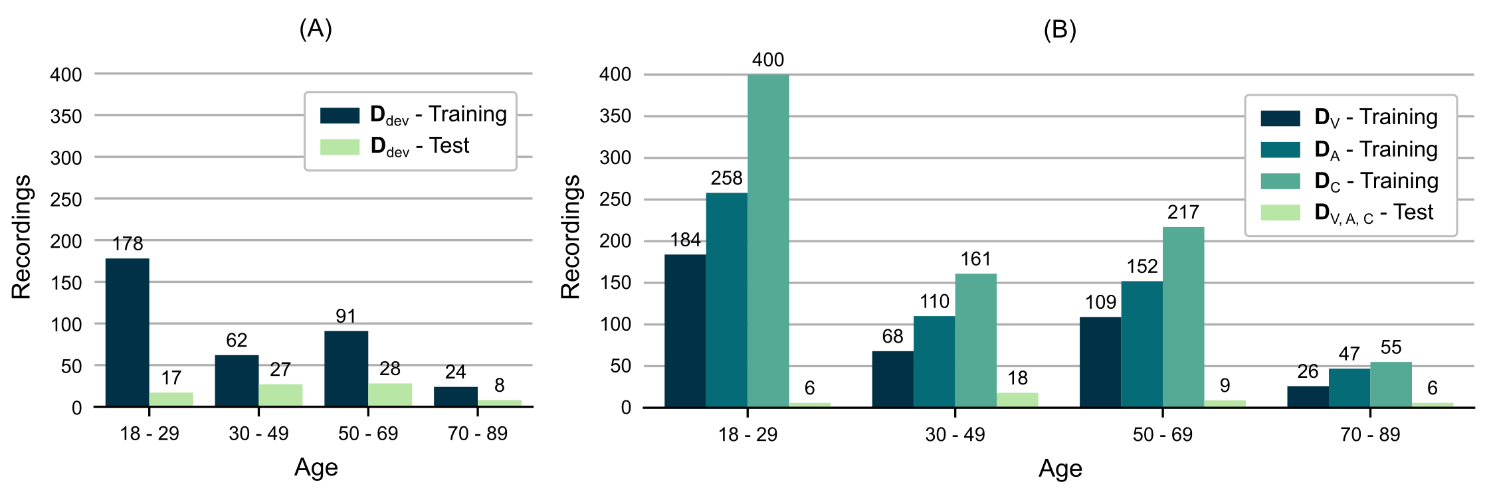
**

**Supplementary Figure 1.** Age distributions for all training and test sets used in (A) HSV model development (see Section 2.2) and (B) evaluation of multi-sensor approaches (see Section 2.3).

**
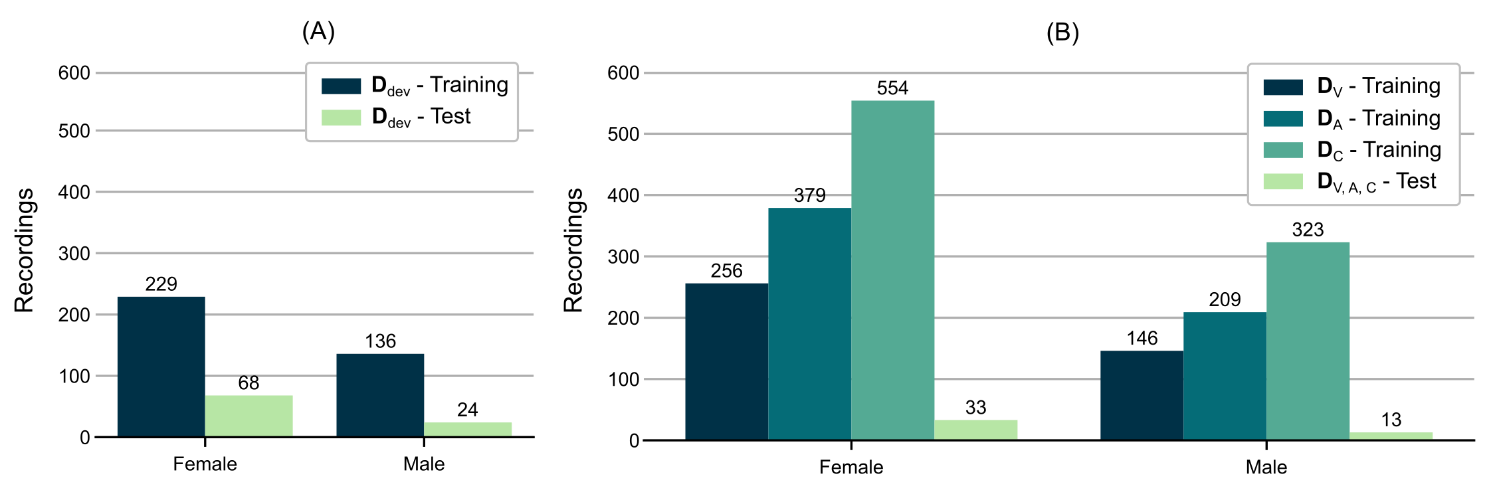
**

**Supplementary Figure 2.** Sex distributions for all training and test sets used in (A) HSV model development (see Section 2.2) and (B) evaluation of multi-sensor approaches (see Section 2.3).

# Model hyperparameters

**Supplementary Table 1.** Hyperparameter grids used in model development. For each model, the hyperparameter value ranges were narrowed down through preliminary experiments to minimize the time and computational cost of the grid search. Default values were applied to any hyperparameters not listed here. If the class weight could not be defined via the hyperparameters, a class weight inversely proportional to the class size was applied via sample weights during model training. The hyperparameters of the final models (XGBoost for the HSV model; LR for the acoustic and clinical models) are indicated in bold type. Note that the selected hyperparameters for the acoustic and clinical models are identical.

| Model | Class | Hyperparameter grid |
| --- | --- | --- |
| **LR** | [sklearn.linear_model.LogisticRegression](https://scikit-learn.org/stable/modules/generated/sklearn.linear_model.LogisticRegression.html) | penalty: [**‘l2’**]  C: [0.005, 0.01, **0.05**, 0.1, 0.25, 0.5]  solver: [**'lbfgs'**, 'liblinear']  max_iter: [**10000**]  class_weight: [**'balanced'**] |
| SVM (linear) | [sklearn.svm.SVC](https://scikit-learn.org/stable/modules/generated/sklearn.svm.SVC.html#sklearn.svm.SVC) | C: [0.01, 0.05, 0.1, 0.5, 1, 5]  kernel : ['linear']  gamma: ['scale', 'auto']  max_iter: [-1]  probability: [True],  class_weight: ['balanced'] |
| SVM (RBF) | [sklearn.svm.SVC](https://scikit-learn.org/stable/modules/generated/sklearn.svm.SVC.html#sklearn.svm.SVC) | C: [0.01, 0.05, 0.1, 0.5, 1, 5]  kernel : ['rbf']  gamma: ['scale', 'auto']  max_iter: [-1]  probability: [True],  class_weight: ['balanced'] |
| DT | [sklearn.tree.DecisionTreeClassifier](https://scikit-learn.org/stable/modules/generated/sklearn.tree.DecisionTreeClassifier.html#sklearn.tree.DecisionTreeClassifier) | max_depth: [2, 4, 8, 16, 32]  min_samples_split: [2, 8, 16, 32, 64]  min_samples_leaf: [1, 8, 16, 32, 64]  max_features: ['sqrt', 'log2']  max_leaf_nodes: [2, 4, 8, 16, 32]  class_weight: ['balanced'] |
| AdaBoost | [sklearn.ensemble.AdaBoostClassifier](https://scikit-learn.org/stable/modules/generated/sklearn.ensemble.AdaBoostClassifier.html#sklearn.ensemble.AdaBoostClassifier) | n_estimators: [32, 64, 128]  learning_rate: [0.01, 0.1, 1] |
| LogitBoost | [logitboost.LogitBoost](https://logitboost.readthedocs.io/index.html) | n_estimators: [32, 64, 128, 256]  learning_rate: [0.01, 0.1, 1] |
| LGBM | [lightgbm.LGBMClassifier](https://lightgbm.readthedocs.io/en/latest/pythonapi/lightgbm.LGBMClassifier.html#lightgbm.LGBMClassifier) | num_leaves: [2]  max_depth: [1]  learning_rate: [0.1]  n_estimators: [32, 64, 128]  objective: ['binary']  min_child_samples: [20]  metric: ['binary_logloss']  class_weight: ['balanced'] |
| **XGBoost** | [xgboost.XGBClassifier](https://xgboost.readthedocs.io/en/release_3.0.0/python/python_api.html#xgboost.XGBClassifier) | n_estimators: [32, 64, **128**]  max_depth: [**1**]  max_leaves: [**2**]  learning_rate: [**0.1**]  objective: [**'binary:logistic'**]  eval_metric: [**'logloss'**] |
| CatBoost | [catboost.CatBoostClassifier](https://catboost.ai/docs/en/concepts/python-reference_catboostclassifier) | iterations: [32, 64, 128]  depth: [1]  max_leaves: [2]  learning_rate: [0.05, 0.1]  grow_policy: ['Lossguide']  l2_leaf_reg: [3, 15, 30]  eval_metric: ['Logloss']  loss_function: ['Logloss']  auto_class_weights: ['SqrtBalanced'] |
| TabNet | [pytorch_tabnet.tab_model.TabNetClassifier](https://dreamquark-ai.github.io/tabnet/generated_docs/pytorch_tabnet.html#module-pytorch_tabnet.tab_model) | n_d: [8, 16, 32, 64]  n_steps: [3, 6, 10] |

# Clinical model selection

**Supplementary Table 2.** Classification results of the model selection performed on the clinical database $\boldsymbol{D}_{C}^{train}$ (see Section 2.3.1). For each model, results are reported in terms of mean and standard deviation of the 5-fold CV. The selected model is highlighted in bold type.

| Model | Accuracy | Sensitivity | Specificity |
| --- | --- | --- | --- |
| **LR** | **0.802 ± 0.031** | **0.761 ± 0.112** | **0.809 ± 0.036** |
| SVM (linear) | 0.787 ± 0.046 | 0.768 ± 0.133 | 0.791 ± 0.048 |
| SVM (RBF) | 0.780 ± 0.041 | 0.884 ± 0.068 | 0.761 ± 0.044 |
| DT | 0.762 ± 0.061 | 0.826 ± 0.132 | 0.750 ± 0.074 |
| RF | 0.764 ± 0.041 | 0.899 ± 0.069 | 0.739 ± 0.046 |
| AdaBoost | 0.741 ± 0.051 | 0.913 ± 0.032 | 0.709 ± 0.059 |
| LogitBoost | 0.872 ± 0.027 | 0.439 ± 0.085 | 0.953 ± 0.020 |
| LGBM | 0.777 ± 0.025 | 0.876 ± 0.065 | 0.758 ± 0.034 |
| XGBoost | 0.774 ± 0.032 | 0.884 ± 0.078 | 0.754 ± 0.042 |
| CatBoost | 0.854 ± 0.041 | 0.651 ± 0.149 | 0.892 ± 0.027 |

# Confusion matrices


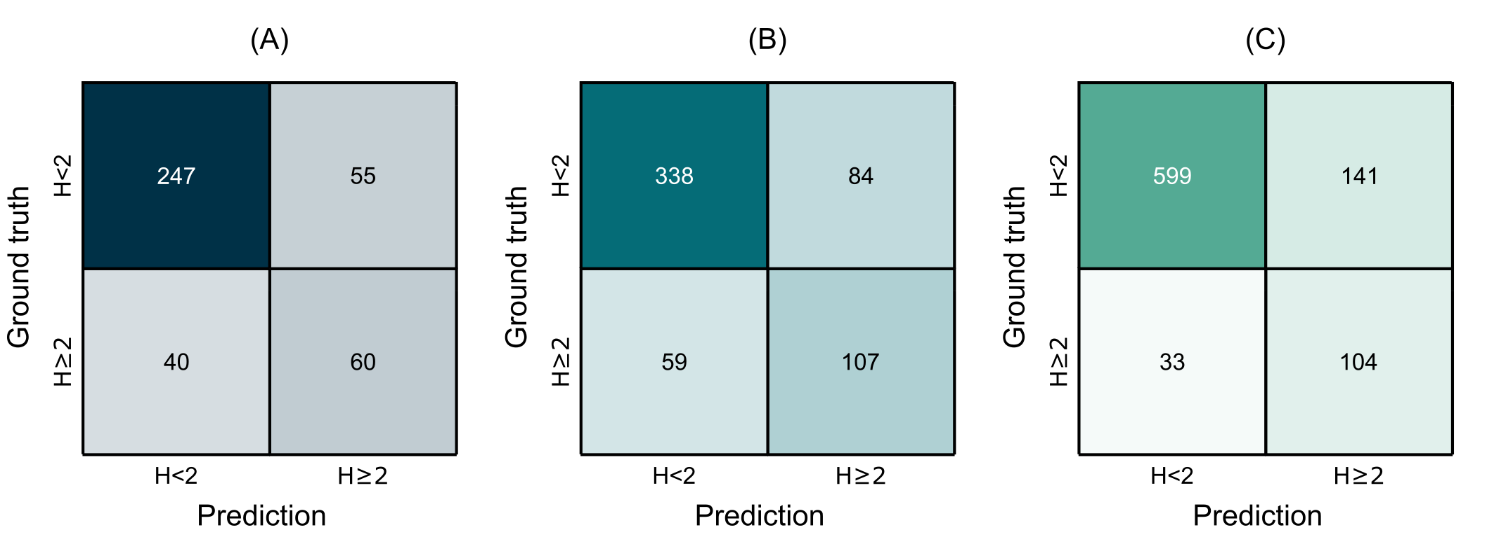


**Supplementary Figure 3.** Confusion matrices showing the classification results of the five-fold CV using (A) $m_{V}$ and $\boldsymbol{D}_{V}^{train}$, (B) $m_{A}$ and $\boldsymbol{D}_{A}^{train}$, and (C) $m_{C}$ and $\boldsymbol{D}_{C}^{train}$.


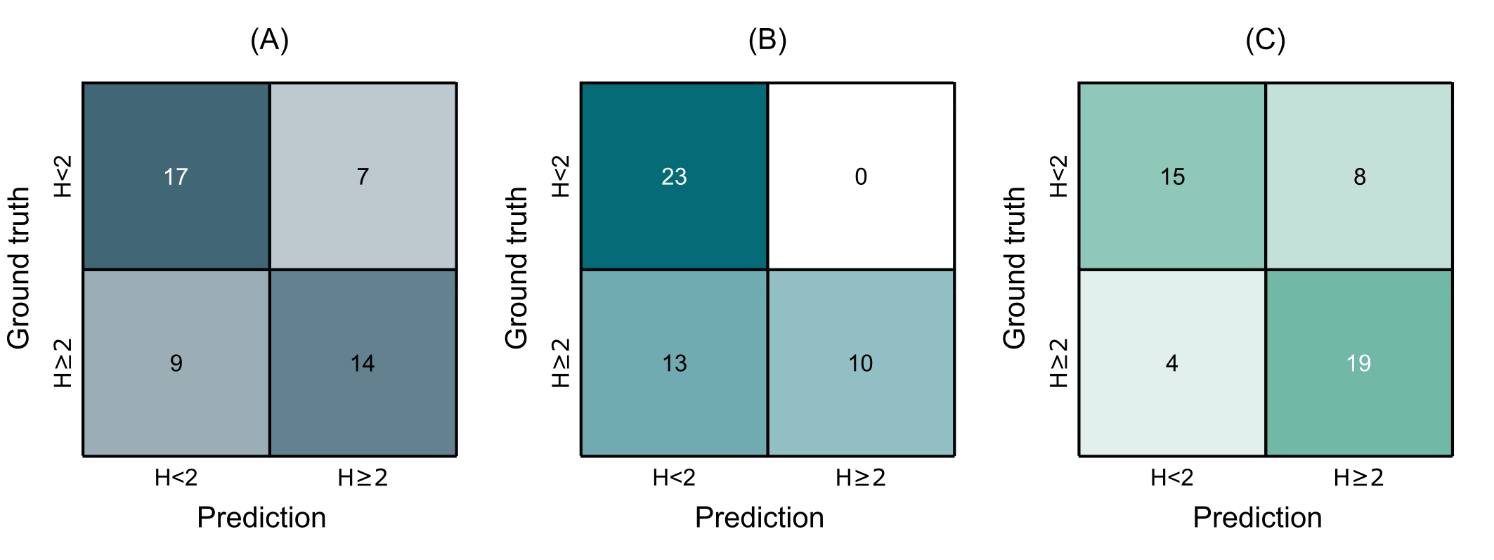


**Supplementary Figure 4.** Confusion matrices showing the classification results on the shared test set using (A) $m_{V}$, (B) $m_{A}$, and (C) $m_{C}$.


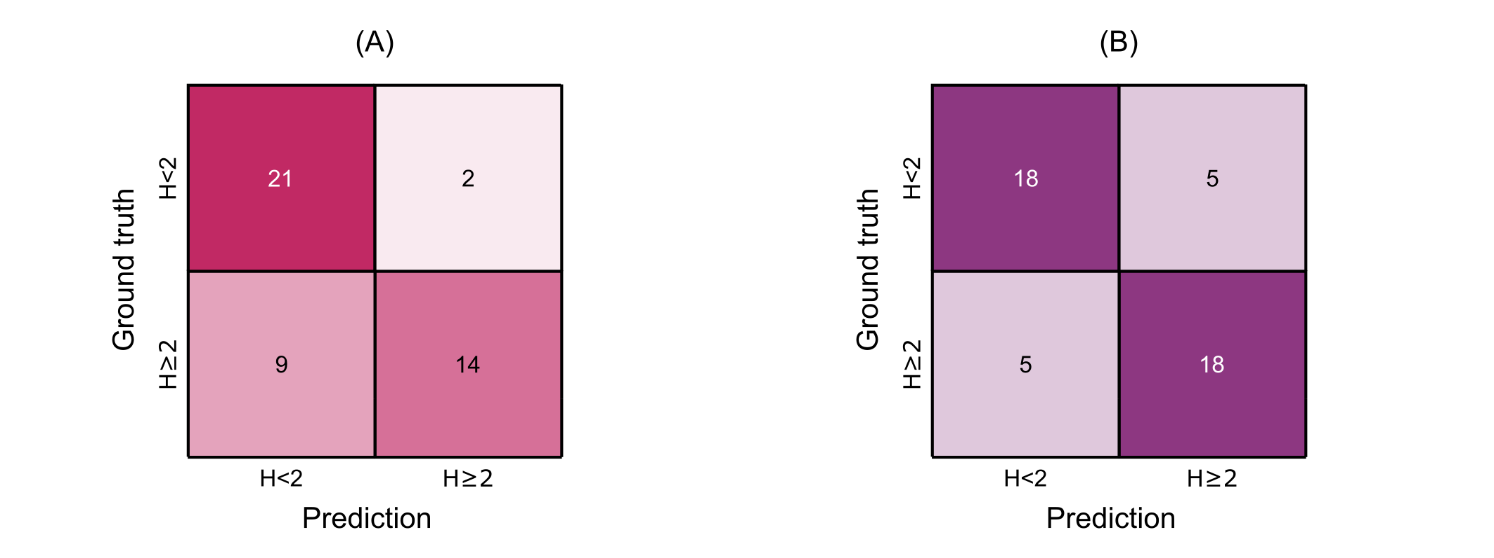


**Supplementary Figure 5.** Confusion matrices showing the classification results on the shared test set using ensemble model (A) $m_{VA}$ and (B) $m_{VAC}$.
